# Mucocutaneous manifestations as early indicators of systemic disease: a narrative review

**DOI:** 10.3389/fmed.2026.1828524

**Published:** 2026-07-30

**Authors:** Sarah Ammar Al Dhamin, Dalia AlDhamin, Zaed Jaber

**Affiliations:** 1 Independent Researcher, Dubai, United Arab Emirates; 2 College of Medicine, Mohammed Bin Rashid University of Medicine and Health Sciences, Dubai Health, Dubai, United Arab Emirates

**Keywords:** autoimmune disease, dermatologic signs, metabolic disease, mucocutaneous manifestations, ocular manifestations, oral lesions, paraneoplastic dermatoses, systemic disease

## Abstract

**Background:**

Mucocutaneous findings may provide important early clues to systemic disease. Because the skin, oral mucosa, eyes, and other visible mucosal surfaces are readily accessible during clinical examination, their careful assessment can contribute to earlier recognition of autoimmune, infectious, metabolic, endocrine, inflammatory, and malignant conditions.

**Objective:**

This narrative review aims to summarize clinically relevant mucocutaneous manifestations that may act as early indicators of systemic disease, with emphasis on their diagnostic significance and practical relevance for clinicians across specialties.

**Methods:**

A narrative literature review was conducted using PubMed/MEDLINE, Scopus, Web of Science, Google Scholar, and reference list screening. Literature was selected to provide a clinically balanced overview of mucocutaneous findings associated with systemic disease, including autoimmune disorders, infectious diseases, metabolic and endocrine disorders, inflammatory conditions, and paraneoplastic syndromes. Priority was given to peer-reviewed clinical studies, reviews, consensus-based sources, and dermatology references that clarified diagnostic relevance, disease associations, or clinical implications.

**Results:**

Mucocutaneous manifestations were identified across several systemic disease categories. In autoimmune disease, findings such as malar rash, photosensitivity, oral ulcers, heliotrope rash, and Gottron papules may support the diagnosis of systemic lupus erythematosus and dermatomyositis. Infectious diseases such as HIV infection and syphilis may present with oral candidiasis, oral hairy leukoplakia, mucous patches, or palmoplantar rash. Metabolic and endocrine disorders may be associated with acanthosis nigricans, eruptive xanthomas, xanthelasma, and other cutaneous markers of insulin resistance, dyslipidemia, or endocrine dysfunction. Paraneoplastic dermatoses, including necrolytic migratory erythema, malignant acanthosis nigricans, dermatomyositis, and acquired ichthyosis, may precede the diagnosis of internal malignancy.

**Conclusion:**

Recognition of mucocutaneous manifestations can support earlier diagnosis of systemic disease and guide appropriate investigation. A structured clinical approach to visible dermatologic and mucosal signs may improve diagnostic accuracy, reduce delays, and promote interdisciplinary collaboration between dermatology, internal medicine, oral medicine, ophthalmology, and primary care.

## Introduction

Mucocutaneous manifestations are often the first to indicate the presence of a systemic disease process. As these areas are examined during visits to various medical specialties, recognition of these findings may aid in diagnosing systemic diseases (1–3).

Various systemic diseases can be associated with specific mucocutaneous manifestations. Autoimmune and connective tissue diseases, including systemic lupus erythematosus and dermatomyositis, may present with clinically important cutaneous and mucosal findings that support diagnosis and guide further evaluation (3–10). Infectious diseases such as human immunodeficiency virus (HIV) infection and syphilis may also present with oral, mucosal, or cutaneous lesions that provide important diagnostic clues (11–15).

Metabolic and endocrine diseases are also associated with visible mucocutaneous markers. Acanthosis nigricans may be associated with insulin resistance, obesity, diabetes mellitus, polycystic ovary syndrome, endocrine disorders, and, less commonly, malignancy (16–18). Xanthomas and xanthelasma may indicate underlying dyslipidemia, familial hypercholesterolemia, or severe hypertriglyceridemia (19–21). In addition, paraneoplastic dermatoses such as necrolytic migratory erythema, malignant acanthosis nigricans, tripe palms, acquired ichthyosis, and paraneoplastic pemphigus may precede or accompany internal malignancy (22–27).

Given that mucocutaneous manifestations can be examined by various medical specialties, clinicians from diverse backgrounds are involved in their detection. Early detection of these manifestations can lead to better management of the underlying disease (28).

This review aims to synthesize clinically relevant mucocutaneous manifestations that may support earlier recognition of systemic disease and guide appropriate diagnostic evaluation.

### What this review adds

Although many mucocutaneous manifestations described in this review are well established, their clinical significance is often discussed within isolated specialties such as dermatology, oral medicine, rheumatology, infectious disease, or oncology. This review brings these findings together using a clinically oriented framework that emphasizes early recognition, diagnostic relevance, and interdisciplinary assessment. The added value of this review lies in organizing mucocutaneous signs according to the systemic disease categories they may indicate, clarifying which findings are diagnostic criteria, disease-associated markers, or warning signs for broader systemic evaluation.

## Methods

### Review design

This article was designed as a narrative review of mucocutaneous manifestations that may serve as early indicators of systemic disease. A narrative review approach was selected because the topic covers a broad range of disease categories, clinical specialties, and study designs. The objective was not to perform meta-analysis or formally pool diagnostic accuracy data, but to provide a clinically useful synthesis of recognizable mucocutaneous patterns and their systemic associations.

### Literature search and source selection

A literature search was conducted using PubMed/MEDLINE, Scopus, Web of Science, Google Scholar, and manual screening of reference lists. The search focused on literature discussing dermatologic, oral, ocular, and mucosal manifestations associated with systemic disease. Search terms included combinations of the following: “mucocutaneous manifestations,” “cutaneous signs of systemic disease,” “oral manifestations of systemic disease,” “ocular manifestations of systemic disease,” “systemic lupus erythematosus skin manifestations,” “dermatomyositis Gottron papules,” “HIV oral lesions,” “secondary syphilis rash,” “acanthosis nigricans systemic disease,” “xanthomas dyslipidemia,” “paraneoplastic dermatoses,” “necrolytic migratory erythema,” and “malignant acanthosis nigricans.”

### Eligibility of literature

Included sources were selected if they discussed mucocutaneous findings with relevance to systemic disease diagnosis, early recognition, disease classification, or clinical management. Priority was given to peer-reviewed clinical studies, cohort studies, observational studies, systematic reviews, narrative reviews, consensus statements, and major dermatology or internal medicine references. Sources focused exclusively on localized skin disease without systemic relevance, animal studies, and articles without clear clinical applicability were excluded.

### Data extraction and synthesis

Relevant information was extracted regarding disease category, mucocutaneous manifestation, associated systemic condition, diagnostic significance, and clinical implications. Findings were organized into major disease groups: autoimmune and connective tissue diseases, infectious diseases, metabolic and endocrine disorders, inflammatory and gastrointestinal disease, and paraneoplastic syndromes. Because of the heterogeneity of included evidence and the broad clinical scope of the topic, findings were synthesized descriptively rather than quantitatively.

### Clinical focus of the review

The review emphasizes mucocutaneous findings that may either precede systemic diagnosis, form part of established diagnostic criteria, suggest disease activity, or prompt further investigation for underlying systemic pathology. Particular attention was given to manifestations with practical diagnostic relevance, including malar rash in systemic lupus erythematosus, Gottron papules and heliotrope rash in dermatomyositis, oral and cutaneous lesions in HIV and syphilis, acanthosis nigricans in metabolic and malignant disease, xanthomas in dyslipidemia, and paraneoplastic dermatoses associated with internal malignancy.

### Quality and interpretation of evidence

**Figure 1:**
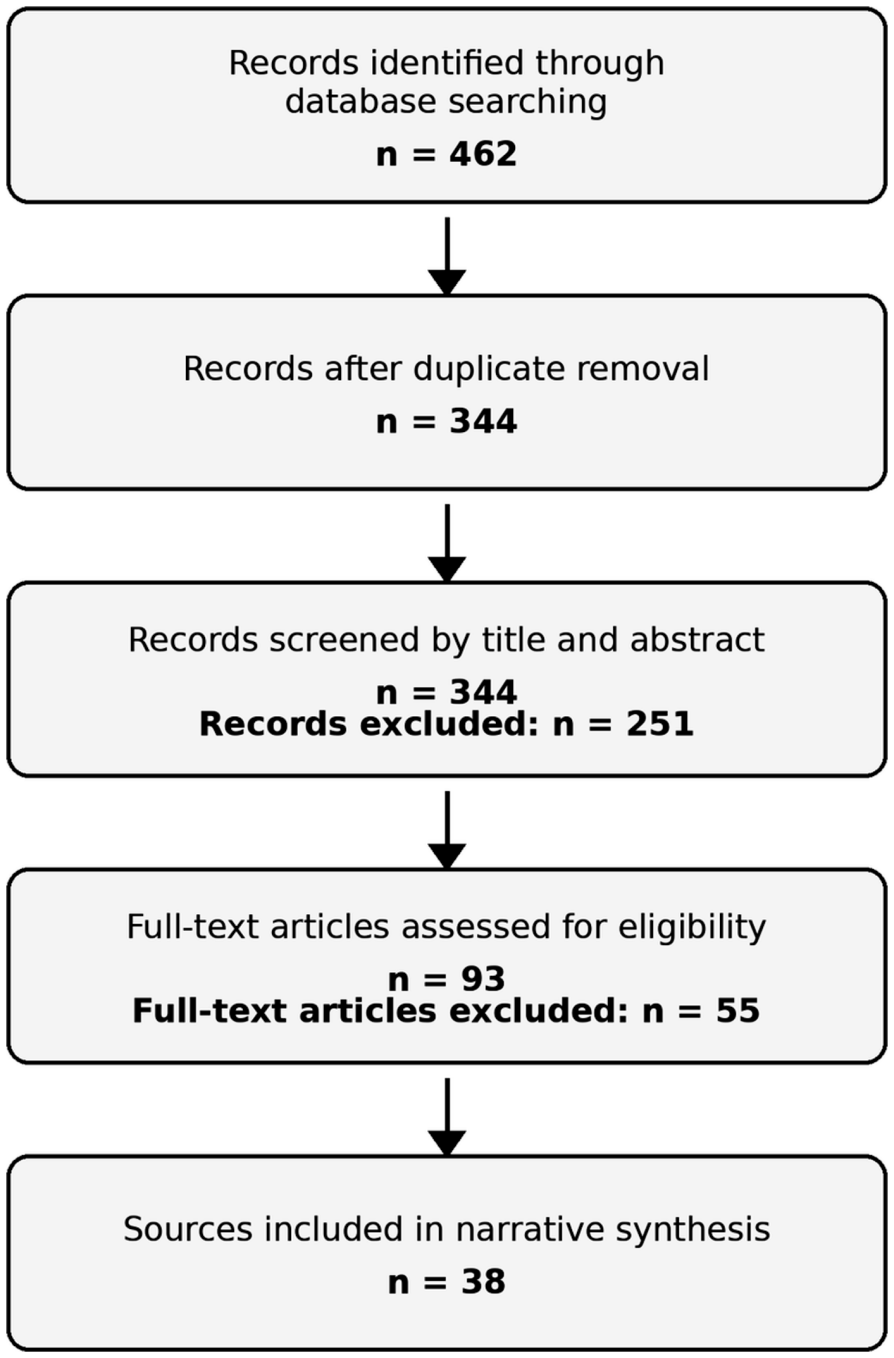
Literature selection flow diagram. This diagram summarizes the process used to identify, screen, and select sources for inclusion in the narrative synthesis. Because this review was revised as a narrative review rather than a systematic review, the diagram is intended to provide transparency regarding literature selection rather than to represent a formal PRISMA-based systematic review process.

Because this article was revised as a narrative review, a formal risk-of-bias assessment was not performed. Instead, the included literature was interpreted according to clinical relevance, consistency across sources, and applicability to early recognition of systemic disease. Greater emphasis was placed on findings supported by established dermatologic, rheumatologic, infectious disease, metabolic, and oncologic literature. Where evidence was limited to case reports, case series, or expert reviews, this limitation is acknowledged in the relevant section (Figure 1).

### Clinical summary of mucocutaneous manifestations

Clinically relevant mucocutaneous manifestations and their systemic associations are detailed in Table 1.

**Table 1:** Clinically relevant mucocutaneous manifestations and their systemic associations.

Disease category	Mucocutaneous manifestation	Associated systemic disease	Clinical significance
Autoimmune/connective tissue disease	Malar rash, photosensitivity, oral ulcers, alopecia	Systemic lupus erythematosus	These findings may support clinical suspicion of SLE and, in some cases, form part of established classification criteria rather than acting only as nonspecific warning signs.
Autoimmune/connective tissue disease	Heliotrope rash, Gottron papules/sign, photosensitive poikiloderma	Dermatomyositis	Gottron papules and heliotrope rash are highly characteristic findings that may precede muscle weakness and should prompt evaluation for inflammatory myopathy and malignancy risk in adults.
Autoimmune/vasculitic disease	Recurrent oral ulcers, genital ulcers, ocular inflammation	Behçet disease	Recurrent mucosal ulceration with ocular or systemic features should raise suspicion for Behçet disease, especially in appropriate clinical and geographic contexts.
Infectious disease	Oral candidiasis, oral hairy leukoplakia, seborrheic dermatitis	HIV infection	These findings may suggest immunosuppression and should prompt HIV testing when clinically appropriate, especially if recurrent, severe, or unexplained.
Infectious disease	Diffuse maculopapular rash involving palms and soles, mucous patches, condyloma lata	Secondary syphilis	Palmoplantar rash and mucosal lesions are important diagnostic clues and should prompt serologic testing for syphilis.
Metabolic/endocrine disease	Acanthosis nigricans	Insulin resistance, obesity, diabetes mellitus, polycystic ovary syndrome, endocrine disease, rarely malignancy	Acanthosis nigricans should not be interpreted only as a marker of insulin resistance; sudden onset, extensive involvement, mucosal disease, or older age at presentation should raise concern for malignant acanthosis nigricans.
Metabolic disease	Eruptive xanthomas, tendon xanthomas, xanthelasma	Dyslipidemia, familial hypercholesterolemia, hypertriglyceridemia	Xanthomas may indicate significant lipid abnormalities and should prompt lipid profile assessment and cardiovascular risk evaluation.
Gastrointestinal/inflammatory disease	Erythema nodosum, pyoderma gangrenosum, aphthous ulcers	Inflammatory bowel disease	These manifestations may precede or accompany intestinal disease and should prompt gastrointestinal assessment when associated with abdominal symptoms, diarrhea, or weight loss.
Paraneoplastic disease	Necrolytic migratory erythema	Glucagonoma syndrome	This characteristic rash may precede recognition of pancreatic neuroendocrine tumor and should prompt evaluation for glucagonoma when associated with diabetes, weight loss, anemia, or stomatitis.
Paraneoplastic disease	Sudden-onset extensive acanthosis nigricans, tripe palms	Gastrointestinal or other internal malignancy	Rapidly progressive, extensive, or mucosal acanthosis nigricans may indicate underlying malignancy and should prompt age-appropriate cancer evaluation.
Paraneoplastic disease	Adult-onset dermatomyositis	Ovarian, lung, gastrointestinal, pancreatic, or other malignancies	In adults, dermatomyositis may be associated with malignancy; clinical context should guide cancer screening.
Paraneoplastic disease	Acquired ichthyosis, Bazex syndrome, paraneoplastic pemphigus	Hematologic or solid malignancy	These less common dermatoses may provide important clues to occult malignancy and warrant systemic evaluation when unexplained.

## Results

### Autoimmune and connective tissue diseases

Autoimmune and connective tissue diseases were among the most clinically recognizable categories associated with mucocutaneous findings. In systemic lupus erythematosus, cutaneous and mucosal manifestations may include malar rash, photosensitivity, oral ulcers, alopecia, discoid lesions, and other lupus-specific or lupus-nonspecific skin findings (3–6). These manifestations should not be interpreted only as nonspecific warning signs, as several are incorporated into established classification frameworks and may provide important diagnostic support when combined with systemic features and serological testing (3).

Dermatomyositis is another condition in which cutaneous findings may be highly characteristic and may precede muscle symptoms. Heliotrope rash and Gottron papules or Gottron sign are particularly important because they may prompt evaluation for inflammatory myopathy even before overt proximal muscle weakness is recognized (7–10). In adults, dermatomyositis also has clinical relevance because of its association with malignancy, making recognition of these cutaneous findings important for both autoimmune and oncologic evaluation (7, 22).

Behçet disease commonly presents with recurrent oral ulceration and may also involve genital ulcers, ocular inflammation, and cutaneous lesions (28–30). Recurrent mucosal ulceration, particularly when accompanied by ocular or systemic features, should raise suspicion for Behçet disease and prompt further assessment. Vitiligo may also be associated with autoimmune comorbidity, particularly autoimmune thyroid disease, and thyroid assessment may be considered when clinically indicated (31, 32).

### Infectious diseases

Several infectious diseases may present with mucocutaneous findings that provide important diagnostic clues. In HIV infection, oral candidiasis, oral hairy leukoplakia, seborrheic dermatitis, and other mucosal or cutaneous manifestations may suggest underlying immunosuppression, particularly when lesions are recurrent, severe, persistent, or unexplained (11–13). Recognition of these findings can support timely HIV testing and earlier linkage to care.

Secondary syphilis is classically associated with diffuse mucocutaneous findings, including a maculopapular rash that may involve the palms and soles, mucous patches, and condyloma lata (14, 15). Because these manifestations can mimic other dermatologic or viral illnesses, awareness of their diagnostic significance is important. Palmoplantar rash or unexplained mucosal lesions should prompt sexual history-taking and serologic testing when clinically appropriate.

### Metabolic and endocrine disorders

Metabolic and endocrine disorders may be associated with visible mucocutaneous signs that provide clues to underlying systemic disease. Acanthosis nigricans is commonly associated with insulin resistance, obesity, diabetes mellitus, polycystic ovary syndrome, and other endocrine abnormalities (16–18). However, it should not be described only as a marker of insulin resistance. Sudden onset, rapid progression, extensive involvement, mucosal involvement, or presentation in an older adult should raise concern for malignant acanthosis nigricans and prompt evaluation for occult malignancy (16–18, 22–24).

Xanthomas and xanthelasma may indicate underlying dyslipidemia, including familial hypercholesterolemia or severe hypertriglyceridemia (19–21). Tendon xanthomas are particularly important because they may reflect inherited lipid disorders and increased cardiovascular risk. Recognition of these findings should prompt lipid profile assessment and cardiovascular risk evaluation.

### Gastrointestinal and inflammatory disease

Inflammatory bowel disease may be associated with extraintestinal mucocutaneous manifestations, including erythema nodosum, pyoderma gangrenosum, and aphthous ulceration (33–35). These findings may occur before, during, or after gastrointestinal symptoms and may correlate with systemic inflammatory activity. When these lesions occur alongside abdominal pain, chronic diarrhea, rectal bleeding, weight loss, or anemia, gastrointestinal evaluation should be considered.

### Paraneoplastic dermatoses

Paraneoplastic dermatoses are clinically important because they may precede or accompany the diagnosis of internal malignancy (22–24). Necrolytic migratory erythema is strongly associated with glucagonoma syndrome and may present with erythematous, erosive, or crusted plaques, often with systemic features such as diabetes, weight loss, anemia, or stomatitis (25–27). Recognition of this pattern can provide an important visual clue to pancreatic neuroendocrine tumor.

**Figure 2:**
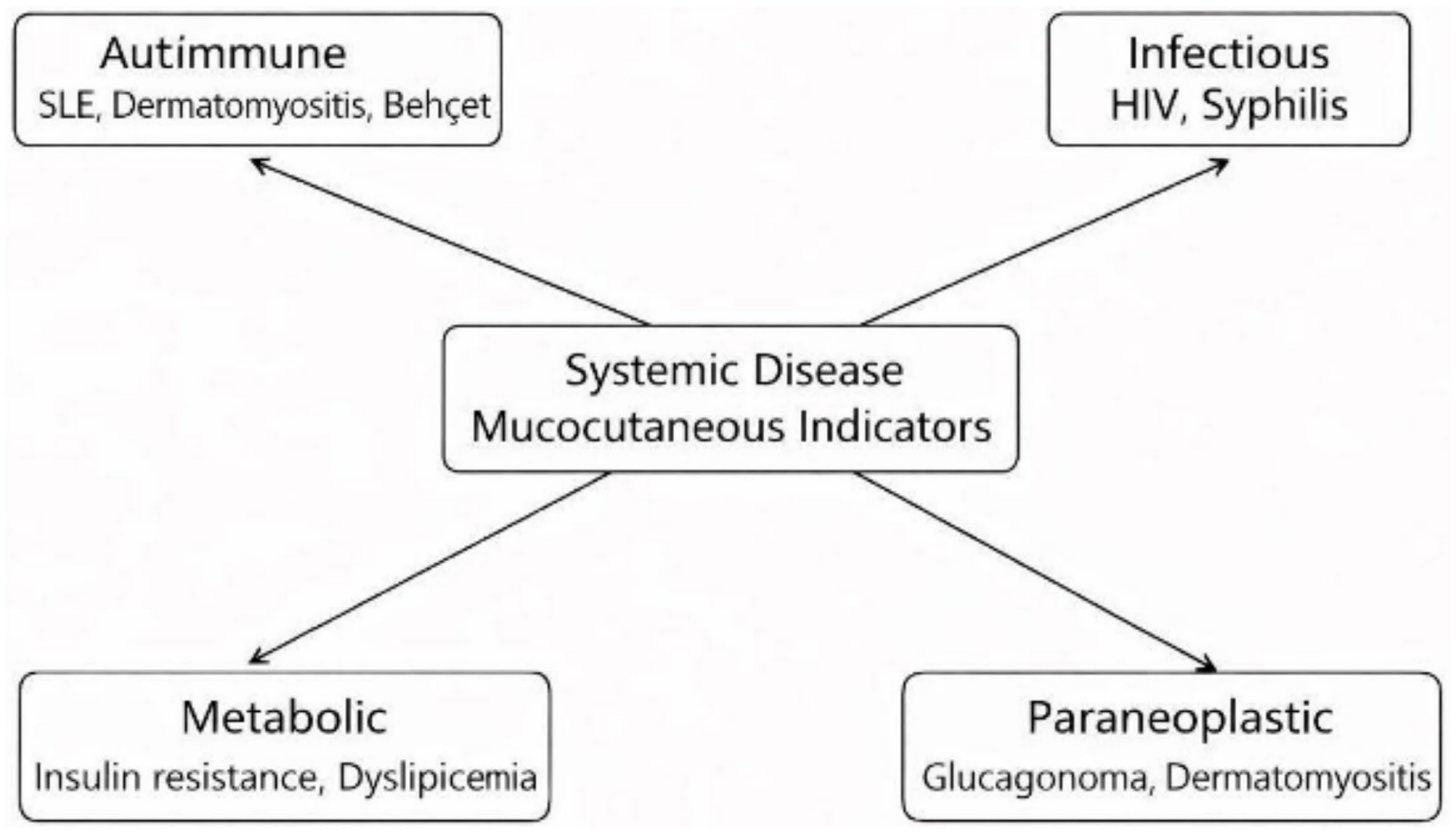
The following figure illustrates the relationship between systemic diseases and the manifestations that occur in the mucocutaneous tissues.

Other paraneoplastic manifestations include malignant acanthosis nigricans, tripe palms, acquired ichthyosis, Bazex syndrome, paraneoplastic pemphigus, and adult-onset dermatomyositis (22–24). These conditions vary in specificity and strength of association with malignancy, but sudden onset, rapid progression, unusual distribution, treatment resistance, or systemic symptoms should prompt further investigation. A structured approach to these findings may improve early detection of occult malignancy (Figure 2).

### Conceptual overview of mucocutaneous manifestations associated with systemic disease

Systemic diseases associated with mucocutaneous findings can be categorized into four major groups:

#### Autoimmune diseases

Examples include systemic lupus erythematosus, dermatomyositis, and Behçet disease. Common manifestations include malar rash, heliotrope rash, Gottron papules, and recurrent oral ulcers.

#### Infectious diseases

Examples include HIV infection and syphilis.

Manifestations include oral candidiasis, oral hairy leukoplakia, mucous patches, and diffuse cutaneous rash.

#### Metabolic and endocrine disorders

Examples include insulin resistance and dyslipidemia.

Characteristic findings include acanthosis nigricans, xanthomas, and xanthelasma.

#### Paraneoplastic syndromes

Examples include dermatomyositis-associated malignancy and glucagonoma.

Manifestations include necrolytic migratory erythema and sudden-onset acanthosis nigricans.

## Discussion

### Principal findings

This narrative review highlights the clinical value of mucocutaneous manifestations as visible indicators of systemic disease. The findings reviewed suggest that dermatologic, oral, ocular, and mucosal signs may contribute to earlier recognition of autoimmune, infectious, metabolic, inflammatory, and malignant conditions. Importantly, these manifestations should not be interpreted uniformly as nonspecific clues. Some, such as malar rash in systemic lupus erythematosus and Gottron papules in dermatomyositis, have major diagnostic significance, while others, such as acanthosis nigricans or xanthomas, may function as markers that prompt metabolic or malignancy-related evaluation (3, 7, 16–21).

Across disease categories, the clinical value of mucocutaneous findings lies in pattern recognition and appropriate interpretation within the broader clinical context. For example, oral candidiasis in an otherwise healthy patient may prompt assessment for immunosuppression, while palmoplantar rash with mucous patches may raise suspicion for secondary syphilis (11–15). Similarly, sudden-onset extensive acanthosis nigricans or necrolytic migratory erythema should not be treated as isolated dermatologic disease without considering the possibility of underlying malignancy (16–18, 22–27).

### Clinical implications

Recognition of mucocutaneous signs can help reduce diagnostic delay by guiding targeted history-taking, physical examination, laboratory testing, imaging, and specialist referral. In clinical practice, visible findings should be interpreted according to their morphology, distribution, onset, progression, associated systemic symptoms, and patient risk factors.

A practical approach is to consider whether the finding is: first, part of an established diagnostic or classification framework, such as malar rash in systemic lupus erythematosus or Gottron papules in dermatomyositis (3, 7); second, a marker of systemic immune or infectious disease, such as oral candidiasis in HIV infection or palmoplantar rash in secondary syphilis (11–15); third, a metabolic clue, such as acanthosis nigricans or xanthomas (16–21); or fourth, a possible paraneoplastic warning sign, such as necrolytic migratory erythema, malignant acanthosis nigricans, tripe palms, or adult-onset dermatomyositis (22–27).

This structured interpretation may improve communication between dermatology, internal medicine, rheumatology, infectious disease, endocrinology, oncology, oral medicine, ophthalmology, and primary care.

### Interdisciplinary relevance

Mucocutaneous manifestations may be encountered in multiple clinical settings, including primary care, dermatology, oral medicine, ophthalmology, rheumatology, infectious disease, endocrinology, gastroenterology, and oncology. Because patients may initially present to different specialties depending on the dominant symptom, interdisciplinary awareness is essential. Early recognition of visible signs can support appropriate referral and reduce delays in diagnosing systemic disease.

### Diagnostic delay

Diagnostic delay may occur when mucocutaneous signs are treated as isolated local disease rather than possible manifestations of systemic pathology. This is particularly relevant when findings are recurrent, severe, unexplained, treatment-resistant, rapidly progressive, or associated with constitutional symptoms. Improved clinician awareness of high-yield mucocutaneous patterns may help identify patients who require further systemic evaluation (36, 37).

### Future research

Future research should evaluate the diagnostic accuracy, predictive value, and clinical utility of specific mucocutaneous manifestations in different patient populations. Studies that assess how early recognition of these findings affects referral patterns, diagnostic delay, and patient outcomes would help clarify their practical value in clinical care.

### Limitations

This review has several limitations. First, because it is a narrative review, it does not provide a formal quantitative synthesis, pooled diagnostic accuracy estimates, or meta-analysis. Second, the included literature is heterogeneous and includes different study designs, disease categories, and clinical specialties. Third, some mucocutaneous findings are well-established diagnostic features rather than novel markers; therefore, this review focuses on clinical recognition and diagnostic integration rather than discovery of new disease associations. Fourth, older references were used selectively when they provided foundational descriptions of classic mucocutaneous manifestations; however, more recent literature was prioritized where available. Finally, the diagnostic value of individual mucocutaneous findings varies by clinical context, and these signs should be interpreted alongside history, examination, laboratory testing, imaging, and specialist evaluation.

## Conclusion

Mucocutaneous manifestations can provide important visible clues to systemic disease. Their clinical significance varies: some findings are part of established diagnostic frameworks, some indicate systemic inflammation or infection, and others may serve as metabolic or paraneoplastic warning signs. Recognition of patterns such as malar rash, Gottron papules, oral candidiasis, palmoplantar rash, acanthosis nigricans, xanthomas, necrolytic migratory erythema, and malignant acanthosis nigricans can support earlier investigation and diagnosis. A structured and interdisciplinary approach to these findings may improve diagnostic accuracy, reduce delays, and guide appropriate management.

## Data Availability

The original contributions presented in the study are included in the article/supplementary material, further inquiries can be directed to the corresponding author.
